# Characterization and comparative analysis of the plastome sequence from *Justicia ventricosa* (Lamiales: Acanthaceae)

**DOI:** 10.1080/23802359.2021.1922099

**Published:** 2021-09-14

**Authors:** Junchen Zhou, Qing Du, Mei Jiang, Shengyu Liu, Liqiang Wang, Haimei Chen, Bin Wang, Chang Liu

**Affiliations:** a Institute of Medicinal Plant Development, Chinese Academy of Medical Sciences, Peking Union Medical College, Beijing, China; b College of Pharmacy, Xiangnan University, Chenzhou, China; c Key Laboratory for Qinghai-Tibet Plateau Phytochemistry of Qinghai Province, College of Pharmacy, Qinghai Nationalities University, Xining, China; d Institute of Medical Information & Library, Chinese Academy of Medical Sciences & Peking Union Medical College, Beijing, China; e College of Pharmacy, Heze University, Heze, PR China

**Keywords:** *Justicia*, plastome, hypervariable region, selective pressures analysis, DNA barcode, phylogenetic analysis

## Abstract

*Justicia ventricosa* is a characteristic ethnic herb commonly used to treat Orthopedic pains. Here, to confirm its phylogenetic position and to develop molecular markers that can distinguish different *Justicia* species, we obtained and analyzed the plastome of *Justicia ventricosa.* The plastome was sequenced using the Illumina HiSeq sequencing platform, assembled with NOVOPlasty, and annotated with CPGAVAS2. The genome has a circular structure of 149,700 bp, containing a large single-copy region of 82,324 bp, a small single-copy region of 17,260 bp, and two reverse repeat regions of 25,058 bp each. It encodes 112 unique genes, including 76 protein-coding genes, eight ribosomal RNA genes, and 28 transfer RNA genes. Twenty cis-splicing genes were found. In total, we predicted 19 microsatellite repeats and 13 tandem repeat sequences. For distributed repeats, four were palindrome repeats and five were direct repeats. To find the highly variable intergenic spacer (IGS) regions, we calculated the K2P distances for IGS regions from four *Justicia* species. The K2P values ranged from 6.11 to 57.82. The largest K2P distances were found for *ccs*A*-ndh*D, *pet*B*-pet*D, *psb*K*-psb*I, and *ycf*4*-cem*A. Phylogenetic analysis results showed that *J. ventricosa* was most closely related to *J. leptostachya*. To determine how *Justicia* species adapt to the environment, we performed selection pressure analysis. Nine genes were found to have undergone positive selection. Lastly, we performed a genome-wise DNA barcode prediction, seven pairs of primers were found. The results provide valuable information that can be used for molecular marker development and bioprospecting in *Justicia* species.

## Introduction

*Justicia ventricosa* Wallich belongs to the Asteraceae family. It is found in the South and Southwest regions of China, including Guangdong, Guangxi, Yunnan, Hainan provinces, and Hong Kong SAR, and it is also distributed in Vietnam to Thailand and Myanmar. It is usually found under the sparse forest or shrub near the village, which are both wild and cultivated (Khaing et al., 2020). Some *Justicia* species are valuable medicines (Lino et al. 1997; Kitadi et al. 2019). The whole plant of *J. ventricosa* can be used as a medicine, which can reinforce tendons and bones, dispel wind and dampness, and treat fracture, sprain, arthritis, chronic low back, and leg pain (Gomez et al. 1996).

The complete plastome can be a circular, linear, or polycyclic double-stranded DNA molecule, with the length mostly around 120–160 kb (Bock 2015). In most higher plants, the complete plastome is highly conserved and consists of four parts, including a small single-copy (SSC) region, a large single-copy (LSC) region, and two inverted repeats (IRA and IRB) regions. The complete plastome sequence can be used for better barcoding, understanding of phylogenetic relationship, understanding patterns of gene loss, understanding adaptive changes that optimize photosynthesis, and potential application in synthetic biology (Olejniczak et al. 2016). To take advantage of these benefits with a complete plastome sequence, we sequenced and assembled a complete plastome sequence of *J. ventricosa* and compared it with other *Justicia* species.

## Materials and methods

### Plant materials, DNA extraction

Fresh leaf samples of *J. ventricosa* were collected from the Guangxi Medical Botanical Garden, Nanning, Guangxi, China (Geospatial coordinates: N22.859968, E108.383475). The specimen was deposited at the Institute of Medicinal Plant Development with the specimen number implad201910236 (HM Chen, hmchen@implad.ac.cn). Total genomic DNA was extracted from the fresh leaf cells of *J. ventricosa* using the plant genomic DNA kit (Tiangen Biotech, Beijing, China).

### Plastome sequencing, assembly, and annotation

We constructed the library with an insert size of 500 bp fragments. We sequenced the library using the Illumina HiSeq platform (Caporaso et al. 2012). The genome was assembled using NOVOPlasty with default parameters (Dierckxsens et al. 2017) and annotated with CPGAVAS2 web service (Shi et al. 2019). The final genome assembly and annotation results have been submitted to GenBank with the accession number MW580585.

### Genome analysis

The complete plastome sequences of *J. adhatoda* (NC_047476.1), *J. leptostachya* (NC_044668.1), *J. nutans* (NC_042162.1), and *J. pseudospicata* (NC_044862.1), *Silvianthus bracteatus* (NC_044974.1) were downloaded from the GenBank database for further analysis. The IR regions of the four *Justicia* species were analyzed using the IRscope online software. The genetic distance of the intergenic spacer (IGS) was calculated by the distmat program from EMBOSS (v6.3.1) and Kimura 2-parameter (K2P) evolutionary model (Rice et al. 2000; Casanellas et al. 2020). The selection pressure of protein-coding genes (PCGs) was analyzed using the aBSREL (adaptive branch-site random effects likelihood) model implemented in the Hyphy software (Smith et al. 2015). We used ecoPrimers software to identify potential DNA barcoding markers to distinguish the four *Justicia* species (Riaz et al. 2011; Khan et al. 2020).

### Phylogenetic analysis

We performed the phylogenetic analysis with the plastomes of five *Justicia* species and that of *Silvianthus bracteatus* as the outgroup taxon. The shared protein sequences of these species were extracted by PhyloSuite (v1.1.16) and aligned with MAFFT (v7.313) (Katoh et al. 2002; Zhang et al. 2020). We performed the phylogenetic analysis using the maximum-likelihood (ML) method implemented in IQ-TREE (v1.6.8) with the TVM + F+I + G4 nucleotide substitution mode (Nguyen et al. 2015). We assessed the reliability of the phylogenetic tree by bootstrap test with 1000 replications. We generated the final phylogenetic tree with the maximum-likelihood (ML) method implemented in MEAG7.0 (Kumar et al. 2016).

## Results

### Genome organization and compositions

**Figure 1.**
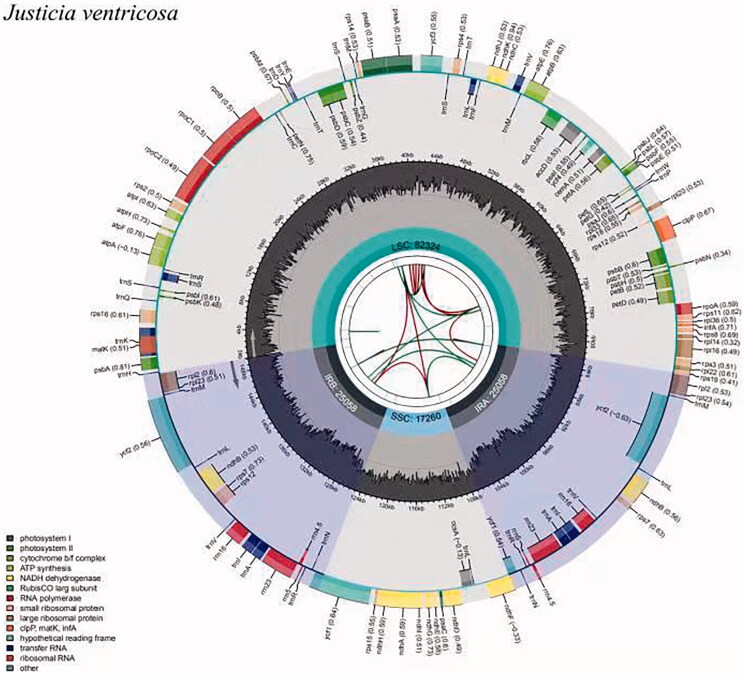
Graphic representation of features identified in *J. ventricose* plastome by using CPGview-RSG (http://www.herbalgenomics.org/cpgview). The map contains seven circles. From the center going outward, the first circle shows the distributed repeats connected with red (the forward direction) and green (the reverse direction) arcs. The next circle shows the tandem repeats marked with short bars. The third circle shows the microsatellite sequences as short bars. The fourth circle shows the size of the LSC and SSC. The fifth circle shows the IRA and IRB. The sixth circle shows the GC contents along the plastome. The seventh circle shows the genes having different colors based on their functional groups.

The plastome sequence of *J. ventricosa* is a typical circular DNA molecule with a total length of 149,700 bp. A schematic representation of the plastome is shown in Figure 1. It has a conserved tetrad structure, including an LSC region, an SSC region, and a pair of IR regions, with length of 82,324 bp, 17,260 bp, and 25,058 bp, respectively. The total GC content in the plastome of *J. ventricosa* is 38.36%. The GC content of the IR region (43.36%) is higher than those of the SSC (32.67%) and LSC (36.52%) regions. The length of the coding sequence (CDS) in the plastome is 79,767 bp, accounting for 53.28% of the whole genome length. The size of the rRNA gene is 9410 bp, accounting for 6.29% of the entire genome length. The tRNA gene's length is 2873 bp, accounting for 1.92% of the total genome length.

### Gene content

**Figure 2.**
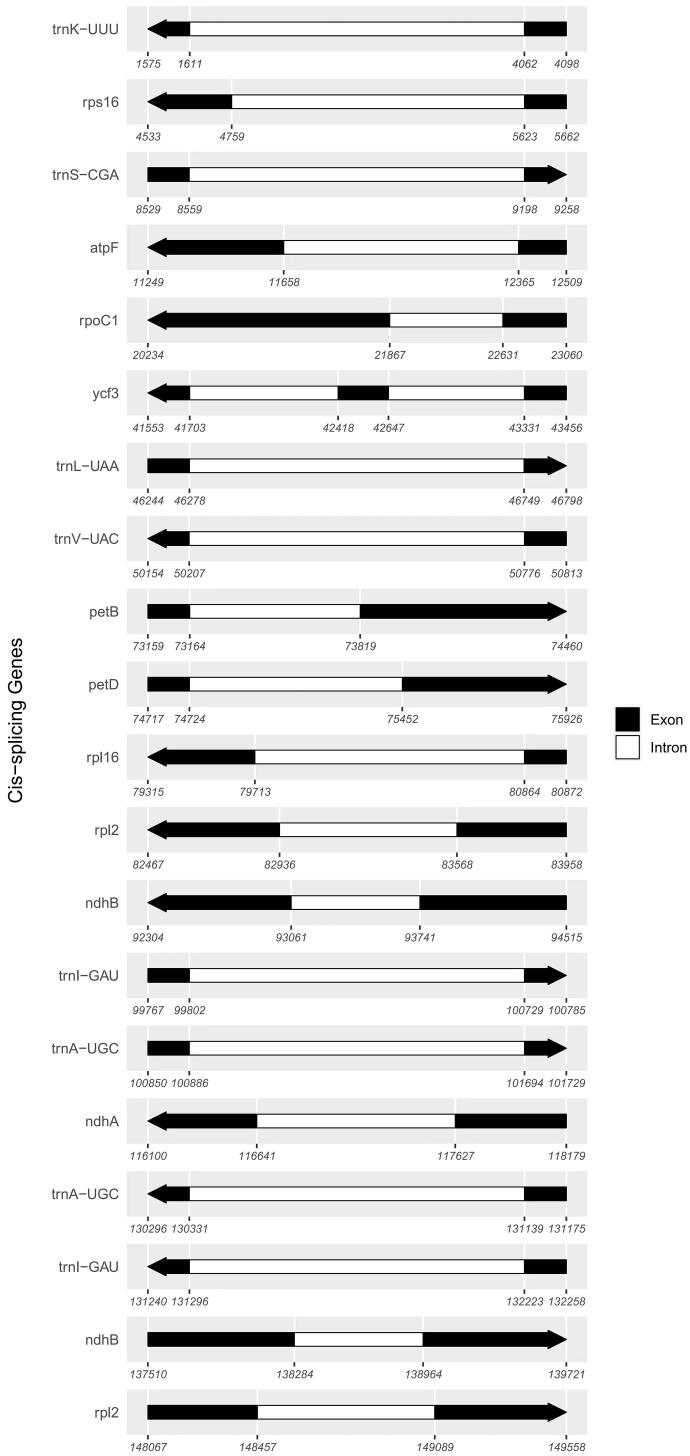
Schematic presentation of the structure of cis-splicing genes from the plastome of *J. ventricosa*. The white area is Intron, and the black area is the exon. The arrow shows the sense direction of the genes.

The plastome of *J. ventricosa* encodes 112 unique genes, including 76 PCGs, 28 tRNA genes, and eight rRNA genes, which were made of two copies of *rrn*16S, *rrn*23S, *rrn*4.5S, and *rrn*5S genes. The gene structures of 20 spliced genes are shown in Figure 2. The black regions represent the exons. The arrow indicates the sense direction. The 20 spliced genes contain 12 PCGs and eight tRNA genes. All PCGs except *ycf*3 have only one intron. In contrast, the *ycf*3 gene contains two introns. Three genes, *pet*B, *pet*D, and *rp*l16, have small exons, consistent with those found in other plastomes (Table 1).

**Table 1.** Genes contents of *J. ventricose* chloroplast genome.

Category of genes	Group of genes	Name of genes
rRNA	rRNA genes	*rrn16S*(×2)*, rrn23S*(×2)*, rrn5S*(×2)*, rrn4.5S*(×2)
tRNA	tRNA genes	28trn genes (6 contains 1 intron)
Self-replication	Small subunit of ribosome	*rps11, rps12*(×2)*, rps14, rps15, rps16, rps18, rps19, rps2, rps3, rps4, rps7, rps7, rps8*
	Large subunit of ribosome	*rpl14, rpl16, rpl2*(×2)*, rpl20, rpl22, rpl23, rpl23, rpl32, rpl33, rpl36*
	DNA dependent RNA polymerase	*rpoA, rpoB, rpoC1, rpoC2*
Photosynthesis	Subunits of NADH-dehydrogenase	*ndhA, ndhB*(×2)*, ndhC, ndhD, ndhE, ndhF, ndhG, ndhH, ndhI, ndhJ, ndhK*
	Subunits of photosystem I	*psaA, psaB, psaC, psaI, psaJ*
	Subunits of photosystem II	*psbA, psbB, psbC, psbD, psbE, psbF, psbI, psbJ, psbK, psbL, psbM, psbN, psbT, psbZ, ycf*3
	Subunits of cytochrome b/f complex	*petA, petB, petD, petG, petL, petN*
	Subunits of ATP synthase	*atpA, atpB, atpE, atpF, atpH, atpI*
	Large subunit of rubisco	*rbcL*
Function unknown	Maturase	*matK*
	Protease	*clpP*
	Envelope membrane protein	*cemA*
	Subunit of Acetyl-CoA-carboxylase	*accD*
	c-type cytochrome synthesis gene	*ccsA*
	Translational initiation factor	*infA*
Genes of unknown functions Open Reading		*ycf1, ycf2*(×2)*, ycf4*

The ‘(×2)’ indicates that the gene located in the IRs and thus had two copies.

### Repeat sequences

Microsatellite repeats are sequences consisting of multiple repeat units with length ranging from 1 to 6 nucleotides. In the plastome of *J. ventricosa*, there were 19 microsatellite repeats (A/T), followed by repeats made up with G/C, AT/AT, and AAG/CTT as repeat units (Table 2). Tandem repeat sequence refers to identical DNA segments lying one after the other in a sequence. It is similar to microsatellite sequence except that the repeat unit is usually ≥7. We identified 13 tandem repeat sequences, whose total length ranged from 25 to 46 bp. The similarity between all repeat units is ≥90% (Table 3). Dispersed repeats are distributed in the genome in a scattered manner. Dispersed repeats can be classified as palindrome repeats and direct repeats. With the threshold of 1e–4, nine dispersed repeats met the requirements. Four of them are palindrome repeats, with the lengths of the repeat units being 41, 39, 34, and 36 bps, respectively. The other five are direct repeats. The lengths of the repeat units being 41, 39, 39, 35, and 32 bps, respectively (Table 4).

**Table 2.** Microsatellites in the *J. ventricosa* plastome.

Type of repeat	Number of repeats
A/T	19
C/G	1
AT/AT	3
AAG/CTT	1

**Table 3.** Tandem repeats in the *J. ventricosa* plastome.

Start–end	Length of repeat (bp)	Copy number of repeat	The final length of repeat (bp)	Matching score of the repeat (%)
1716–1740	12	2.1	25	100
8236–8264	14	2.1	29	100
44,230–44,264	17	2.1	36	94
45,242–45,281	18	2.2	40	100
55,882–55,909	14	2.0	28	100
58,548–58,591	21	2.1	44	91
66,145–66,179	13	2.6	34	90
66,590–66,624	16	2.2	35	100
74,487–74,517	16	2.0	32	93
77,503–77,548	24	1.9	46	90
80,374–80,411	18	2.1	38	100
120,055–120,090	17	2.1	36	100
120,059–120,096	17	2.2	37	90

**Table 4.** Interspersed repeats in the *J. ventricosa* plastome.

Length of repeat unit I (bp)	Start of repeat unit I	Repeat type	Length of repeat unit II (bp)	Start of repeat unit II	*E*-value
41	96,120	D	41	116,676	1.30e–15
41	116,676	P	41	135,863	1.30e–15
39	42,685	D	39	96,122	2.09e–14
39	42,685	D	39	116,678	2.09e–14
39	42,685	P	39	135,863	2.09e–14
34	7733	P	34	44,145	2.18e–09
36	74,919	P	36	74,919	7.57e–09
35	37,692	D	35	39,916	9.43e–07
32	7732	D	32	34,565	4.58e–05

### IR structure analysis of four *Justicia* species

**Figure 3.**
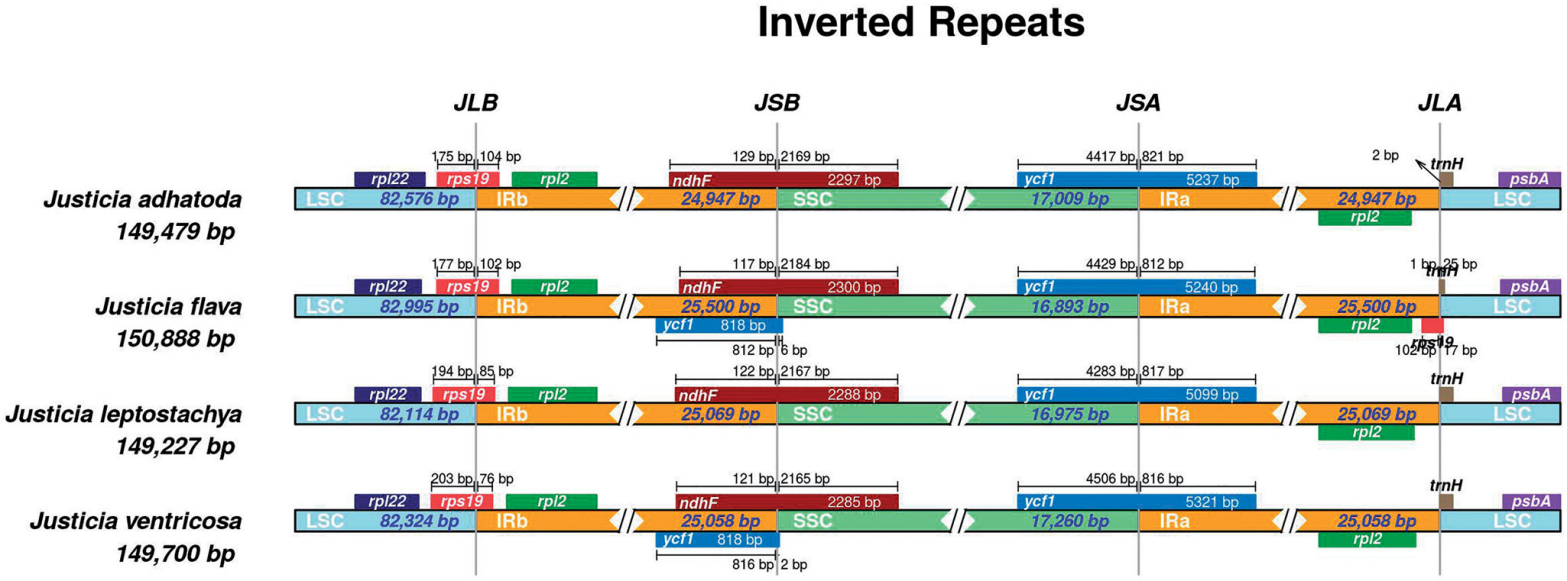
Comparison of LSC, SSC, and IR boundaries of four *Justicia* species. The genes around the border are shown above or below the central horizontal thick line representing the genome. The Latin names and plastome length are shown on the left. JLB, JSB, JSA, and JLA represent the junction sites of LSC/IRb, IRb/SSC, SSC/IRa, and IRa/LSC, respectively. The distances from the start and end of different genes to the junction sites are displayed above and below the corresponding genes.

IR boundary analysis of four complete plastomes of *Justicia* species showed that they had the same boundary structure (Figure 3). For the four species, the *rps*19 genes were located in the border area of LSC and IRb. Its 5′ ends were located in the LSC region, and 3′ ends were located in the IRb region. In contrast, *ndhF* genes were located at the border area of IRb and SSC. Most of the *ycf*1 genes were located in the border area of SSC and IRa. Besides, *rpl*2, *trn*H gene spacers were located in the IRa and LSC border area, respectively. Some of the *ycf*1 genes were found in the border area IRb and SSC in *J. pseudospicata and J. ventricosa.* A small fragment of the *rps*19 gene was found in the border area of IRa and LSC.

### Hypervariable region identification

**Figure 4.**
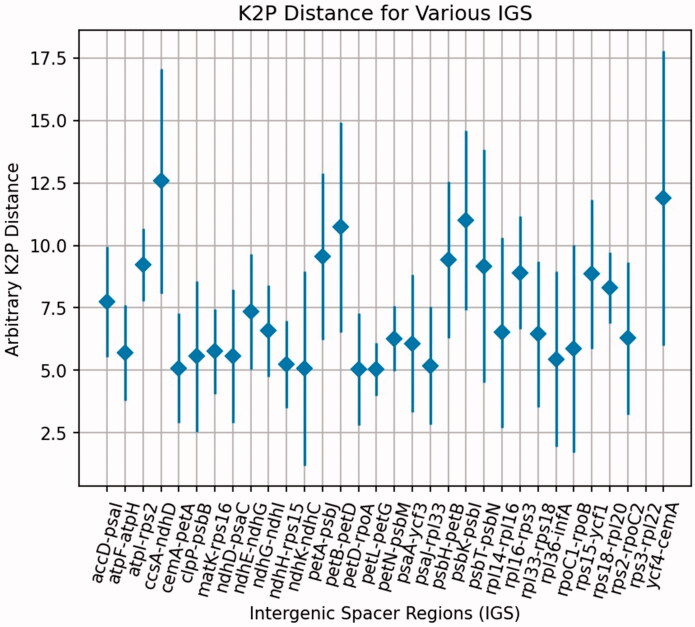
Average K2p distances among the intergenic spacer regions from the four *Justicia* species: *J. adhatoda*, *J. leptostachya*, *J. pseudospicata*, and *J. ventricosa*. The K2p distances were calculated for the corresponding IGS regions pairwisely. The black dots represent the average K2p distances for the corresponding IGS regions.

To find highly variable regions among the *Justicia* plastomes, we calculated the genetic distance of IGS regions using the K2P model. There were 58 IGS regions with K2P values ranging from 6.11 to 57.82. K2P values of *ccs*A-*ndh*D, *pet*B-*pet*D, *psb*K-*psb*I, and *ycf*4-*cem*A were higher, at 12.57, 10.73, 11.00, and 11.89, respectively (Figure 4). The variations of these IGS regions are large, suggesting these regions can be used to develop potential molecular markers for species discrimination.

### Selective pressure analysis

Using the adaptive branch-site random effects likelihood model implemented in the HyPhy software, we analyzed the selection pressure of PCGs in *J. ventricosa*, *J. leptostachya*, *J. adhatoda*, and *J. nutanss*. The genes of *acc*D, *ndh*B, *rpo*A, *rpo*C1 were positively selected in species of *J. ventricosa*. For *J. leptostachya*, three genes of *ndh*F, *rps*12, *ycf*1 were positively selected. Moreover, the genes of *ndh*G, *ycf*2 were found positively selected in *J. adhatoda* and *J. nutanss*, respectively. The optimized branch length, LRT, and *p* value varied in the range of 0.0079–0.0179, 8.1342–21.8014, 0–0.03, respectively. These genes may be related to the adaptation of *J. ventricosa* under diverse environments (Table 5).

**Table 5.** Selection pressure analysis.

Species	Gene	Position	Optimized branch length	LRT	*p* Value
*J. ventricosa*	*acc*D	56,118–57,590	0.0079	21.3902	.0000
	*ndh*B	94,515–92,304		10.4026	.0095
	*rpo*A	76,068–77,081		10.6976	.0082
	*rpo*C1	23,060–20,234		21.8014	.0000
*J. leptostachya*	*ndh*F	107,062–109,350	0.0129	10.5454	.0089
	*rps*12	67,827–67,940 95,117–95,899		8.6738	.0228
	*ycf*1	119,876–124,975		14.9044	.0010
*J. adhatoda*	*ndh*G	114,627–115,157	0.0094	14.7286	.0011
*J. nutans*	*ycf*2	85,633–92,400	0.0179	8.1342	.0300

### Genus-specific DNA barcode identification

To discover the DNA barcode sequences that can distinguish the four species, the plastomes of four *Justicia* species: *J. ventricosa*, *J. leptostachya*, *J. nutans*, *J. adhatoda*, were analyzed using ecoPrimers. The conservative intervals used to design PCR amplification primers are shown in Table 6.

**Table 6.** Primers for amplifying DNA barcodes to distinguish the four *Justicia species*: *J. ventricosa*, *J. leptostachya*, *J. nutans*, and *J. adhatoda*.

No.	Forward primer sequences	Reverse primer sequences
1	AACTGTCCAAAGATGAAAATCTTT GTAATATTCTGAACC	GGATATACAAAAAAGAACCAGTCCT AATGAAAAAGCCGAATAAAT
2	AATGAATTCGGCCTCGGAAACGTTCCGATGTGATCAATTTTT CGTAGGGGTATTGAATAGTTACAGGTAAA	GAACTGAGTTAGCATAGGGAACATATCGAACTATTTATAACTAA TTTGACTTGTTTCTTTCTCTTGTTTG
3	AAGAAATTTCCACCCAAGATTTAATAGT TGGTCCATTCTTAGT	TGCCGATTAGTGTTCCAAAAACTTGACT TCGTTTATTTATATCAAAAAGCTCAG
4	CAAGGTTTTAGCCAGAAAGGAAAAAAGA TTTTTAT	AGGTACATTTAACATACATCTCTCTATTCCAGTCCT TGAAATCTTCCGACCATTCAGGAAGTTGGAATAGTA
5	ATAAATCAGCCCTTCCCGTAGGTTATTTT	AAAATAGGAAAAATATAAATACATACTTTGCCTATGCCTATTTCGAAGAT TTAAACAAAAGGATTCGCAAATAAAAGTGCTAATGCTACAACCAACCCATAAA TCGTTAAAGCTTCCATAAAAGCTAGACTAAGC
6	ATCGTTTTTTGATTTTTATT AAGGGGTTGT	TCAAAGCGTTTGCCCTTATTTCAACAAGGGAAGCAGGGGCTTCCTGAC TAGAAGAACTTTTTTTGCCTTGTGTCCAATTCAATACTAAACAAGTCCGAACTAAT TGAATATTTGTATCAGAAATTCC
7	TCTGTGAGAATAAATCAGAATTCTGGTAGA	ATGTGGATCCTCTCTTGAGAGAAGGAAAATGCGCACTCATTTGATCTTGATC

### Phylogenetic analysis

**Figure 5.**
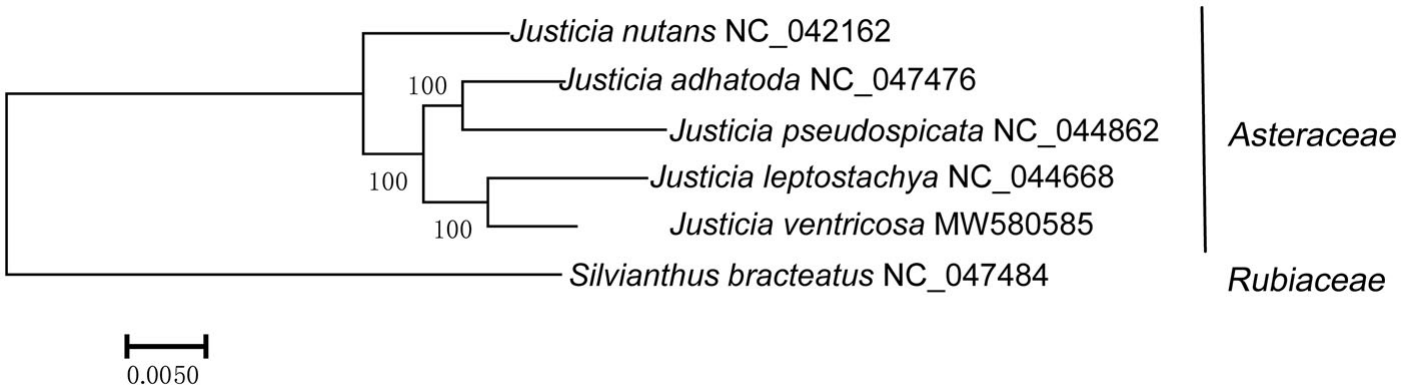
The phylogenetic tree of five *Justicia* species of Acanthaceae and *Silvianthus bracteatus* selected as the outgroup. The tree was constructed using the maximum-likelihood method. Bootstrap support values were calculated from 1000 replicates.

Based on the 42 common protein sequences of five *Justicia* species and *Silvianthus bracteatus*, we constructed the phylogenetic tree (Figure 5). As expected, the *J. ventricosa*, *J. leptostachya*, *J. adhatoda*, *J. nutans*, and *J. pseudospicata* were clustered into one branch. The genetic relationship between *J. ventricosa* and *J. leptostachya*, *J. adhatoda*, and *J. pseudospicata* were the closest, respectively. In contrast, *S. bracteatus* was distantly related to the *Justicia* species. The bootstrap scores of all branches containing *Justicia* species are 100%, supporting the reliability of the tree (Figure 5).

## Discussion

In this study, we obtained and analyzed the plastome of *J. ventricosa* for the first time. Overall, the plastome contains highly conserved features. Some worthy features are the followings. IR boundary analysis of the plastomes of four *Justicia* species shows that the *rps*19 gene is located at the IRB-LSC boundary. Most of the *rps*19 gene sequences are in the LSC region. In contrast, the ycf1 gene is located in the IRB-SSC border. Besides, the K2P values of four IGS regions: *ccs*A-*ndh*D (12.57), *pet*B-*pet*D (10.73), *psb*K-*psb*I (11.00), and *ycf*4-*cem*A (11.89), are high. Highly variable regions can be used as DNA barcodes for species identification. We also identified seven regions that can be used to distinguish the four species: *J. ventricosa*, *J. leptostachya*, *J. nutans*, and *J. adhatoda*. We also performed selective pressure analysis to identify the different genes in the *Justicia* species, which may play an essential role for *Justicia s*pecies to adapt to varied environments (Chen and Blanchette 2007). The phylogenetic tree results showed that the five *Justicia* species are closely related, as would be expected. Overall, there is limited information on the phylogeny of the *Justicia* species (Yaradua et al. 2019). Therefore, further studies on the plastomes of this genus are needed in future studies.

## Data Availability

The data that support the findings of this study are openly available in the GenBank database at https://www.ncbi.nlm.nih.gov with the accession number is MW580585. The associated BioProject, SRA, and Bio-Sample numbers are PRJNA717803, SRR14085608, and SAMN18511406, respectively.
